# Investigation of green supply chain management practices and sustainability in Indian manufacturing enterprises using a structural equation modelling approach

**DOI:** 10.1038/s41598-025-95940-9

**Published:** 2025-04-28

**Authors:** Priya Gupta, Yogesh Sharma, Ajay Chauhan, Bhawna Parewa, Pratibha Rai, Nithesh Naik

**Affiliations:** 1 Atal Bihari Vajpayee School of Management and Entrepreneurship, Jawaharlal Nehru University, New Delhi, 110067 India; 2 University Business School, Karzat, Mumbai, Maharashtra 410201 India; 3 Maharaja Agrasen College, University of Delhi, Delhi, 110096 India; 4 Department of Mechanical and Industrial Engineering, Manipal Institute of Technology, Manipal Academy of Higher Education, Manipal, Karnataka 576104 India

**Keywords:** Green practices, Green logistics, Top management performance, Low carbon performance, Sustainable manufacturing, Sustainable society, Supply chain analytics, Scientific data, Software, Statistics

## Abstract

Green Supply Chain Management (GSCM) has gained increasing attention as a means of ensuring sustainable manufacturing and a sustainable society. This study examines the relationship between GSCM practices and top management performance to understand its effect on low-carbon performance, sustainable manufacturing, and sustainable society. Data were gathered from 389 top-, middle-, and lower-level managers working in bag-manufacturing firms in India. The data were analyzed using a structural equation modelling (SEM) approach. The findings indicate positive and significant relationships among the constructs, with "green product and product design" showing the most substantial influence on "top management performance" (β = 0.274, *p* < 0.001). This top management performance significantly boosts "low carbon performance" (β = 0.375, *p* < 0.001), which in turn positively impacts “sustainable manufacturing” (β = 0.283, *p* < 0.001) and “sustainable society” (β = 0.347, *p* < 0.001). The SEM model explained 20% of the variance in "top management performance," 19.5% in "low carbon performance," 16.1% in "sustainable society," and 14.7% in "sustainable manufacturing." The link between “low-carbon performance” and "Top Management Performance" is found to have a medium effect size, indicating a strong and discernible correlation between the two variables. In practical terms, an organization will likely make significant strides toward sustainability and carbon emissions reduction when senior management actively supports and implements these measures. This study highlights that adopting GSCM practices is limited to improving firm performance and goes beyond creating sustainable manufacturing and society. This study is in the exploratory stage and adopts a holistic approach to understand the impacts of GSCM practices. Further studies on GSCM practices should be conducted to gain deeper insights. The model provides a broader picture for manufacturers to develop a long-term vision while adopting GSCM practices for sustainable manufacturing and sustainability.

## Introduction

The dawn of industrialization saw marked oversight among corporations regarding sustainability and the environmental implications of their business choices. Policies with short-term payoffs were chosen. However, they were damaging to the long-term interests of the people and the planet and were, therefore, not sustainable. To offset short-term expenses, societal and environmental health have been jeopardized by increased long-term hazards. The current consumption rate of individuals and corporations surpasses what our planet can sustainably replenish, leading to dire consequences^1^. The escalation of global warming and surge in CO_2_ levels have reached critical thresholds. Additionally, the swift depletion of resources and environmental decline have caused significant ecological imbalances^2^. Governments worldwide have been compelled to enact policies and implement measures to reduce the detrimental impact of their activities^3,4^. Consequently, companies’ environmental strategies are now a driving force behind their operational and strategic decisions^5^. One prominent supply chain strategy concerning the environment is Green Supply Chain Management (GSCM), which encapsulates upward and downward supply chain elements under the sustainability framework^6^.

GSCM is a cross-disciplinary field that integrates environmental concerns with supply chain management^7^. It considers all the activities involved in the customer order cycle^8^, including product design, procurement, manufacturing, logistics, distribution, and product management at the end of its life cycle. The relevance of GSCM is not only limited to minimizing the negative impacts of industrial activities on the environment, but also ensures that the cost, quality, and performance are not compromised^7^. GSCM has moved beyond the buzzword, and customers and governmental entities have begun to demand that processes, products, and services be more environmentally friendly^9^. Managers must identify and implement environmentally sustainable practices throughout the supply chain. Studies have found that successful trials at the supply chain level also pave the way for an organization’s success^10^. Specifically, in a developing country, the goal of any manufacturer is to strike a balance between its economic and environmental performance^11^. GSCM ensures a balance between economic and environmental performance^12,13^, improves operational and organizational performance^14^.

By far, the manufacturing sector represents the sector that poses the most significant environmental threat. On average, manufacturers are under the utmost pressure to redesign their processes to reduce environmental damage, whether through legislation or even through internal employees, leadership, and perception of risk^15^. Therefore, it is crucial for Indian manufacturers to study the role of GSCM in reorienting their harmful environmental impacts by focusing on supply chain operations and enhancing sustainability performance. Several past studies have focused on how the adoption of GSCM practices by manufacturing firms leads to improved environmental and economic performance, which, in turn, positively impacts operational performance^14^. Therefore, this study examines the role of GSCM in sustainable manufacturing and building a sustainable society in the Indian manufacturing sector. GSCM practices such as "Green Procurement," "Green Logistics," "Regulatory Framework," and "Green Product Design" are considered to measure their contribution to "Top Management Performance," which further contributes to "Low Carbon Performance," which leads to “Sustainable Manufacturing” and "Sustainable Society." After assessing and confirming the reliability and validity of each construct using factor analysis and concluding that the study is free from any bias, a hypothesized conceptual research model using structured equation Modelling was presented and tested.

One significant challenge in supply chain research, particularly when employing survey methods, is the common method bias. This form of bias can inflate or deflate the perceived relationships between variables, potentially leading to misleading conclusions. As^16^ highlighted, acknowledging and addressing common method bias is essential for the integrity and reliability of empirical findings in operations management. This is especially pertinent in studies utilizing single-informant designs, in which the same respondent provides information on both independent and dependent variables. In light of these considerations, our study adopts measures to mitigate the potential influence of common method bias, ensuring a more accurate representation of the relationship between green supply chain practices and firm performance.

While green supply chain management (GSCM) has been recognized as a pivotal factor in enhancing both environmental and organizational performance, the existing literature has often circled around established concepts and frameworks, leaving significant gaps. Schmidt et al.^17^ underscore the paradox in supply chain positioning, suggesting that, while green practices are increasingly adopted, the understanding of their strategic underpinnings remains fragmented. This highlights a critical gap in the extant literature: the need for novel theoretical contributions that can bridge the existing knowledge with new insights into how green supply chain practices are formulated and implemented and their impacts evaluated within diverse organizational contexts. This study seeks to address this gap by proposing a unique theoretical framework that integrates specific new aspects such as a new angle on stakeholder theory, resource-based views, or institutional theory with GSCM. This approach intends to deepen our understanding of the mechanisms through which GSCM practices contribute to sustainable development and competitive advantage, moving beyond conventional narratives. By doing so, we aim to contribute to scholarly discourse with fresh perspectives and a more nuanced understanding of green supply chain dynamics, thereby answering the call for innovative theoretical exploration, as Schmidt et al.^17^ highlight. GSCM serves as a vital strategy for balancing sustainability and operational efficiency. Supply chain analytics (SCA) enhances GSCM by offering data-driven methods to quantify, evaluate, and optimize green practices. For example, predictive analytics enables the modelling of relationships such as the influence of top management performance on low-carbon outcomes, while real-time monitoring tools support actionable decision-making^18,19^.

Zhang and Ruan (2024) examines blockchain’s impact on corporate performance in China, showing its role in improving financial and environmental outcomes through transparency. It highlights variations based on ownership, sensitivity, and technical features, offering insights for businesses and policymakers. Blockchain’s role in sustainable supply chain management, highlighting its potential to improve transparency, efficiency, and sustainability while addressing adoption challenges and proposing solutions for future advancements^20^. The impact of blockchain technology on decision-making in green supply chains under demand uncertainty is worth exploration. A game theory model compares green products with and without blockchain technology, analyzing the optimal introduction of green inputs in a duopoly market. Findings reveal that higher consumer uncertainty can encourage manufacturers to improve eco-friendliness and supply chain performance. However, universal blockchain adoption may compromise product sustainability while boosting supply chain profitability. Blockchain-enabled products are generally priced lower, benefiting consumers, especially when acceptance of sustainable energy is high or uncertain. The study also examines how green supply chain decisions affect system reliability and suggests that feedback control technology can stabilize market competition in dynamic systems.

The study primarily builds on certain foundational works such as Elkington’s Triple Bottom Line (1997) and Porter & van der Linde’s (1995) framework on the competitive advantage of green strategies, stakeholder theory and resource-based view (RBV), integrating them within the context of GSCM. Stakeholder theory explains how organizations must balance diverse stakeholder expectations, particularly regarding environmental sustainability. RBV highlights how sustainable practices can serve as unique organizational resources that deliver a competitive advantage. The paper extends stakeholder theory by demonstrating how GSCM practices align stakeholder interests with sustainable outcomes. It also advances RBV by providing empirical evidence that environmental practices, such as green procurement and logistics, can be leveraged as strategic resources to improve top management performance and overall sustainability metrics. By incorporating structural equation modeling (SEM), the paper empirically validates the theoretical constructs of stakeholder theory and RBV in the GSCM context. This approach helps quantify the impact of green practices on managerial performance, offering insights into operationalizing these theories within manufacturing sectors. This prompts the following research question:

RQ1: How do green supply chain practices adopted by the firms influence the top management practices?

RQ2: What is the relationship between green practices adopted by manufacturing firms and a sustainable environment?

This study provides an overview of the growing significance of Green Supply Chain Management (GSCM), outlines its goals, and diverges from previous research by offering valuable insights into this field of study. Firstly, it examines the relationship between GSCM practices and top management performance. Secondly, it assesses the impact of GSCM practices on low-carbon performance, sustainable manufacturing, and societal sustainability.

The exploration begins with a review of the current literature, development of a theoretical framework, and formulation of hypotheses in Section “Literature review and hypothesis development”. The methodology is detailed in Section “Methodology”, which addresses the questionnaire design, data collection methods, demographic variables, and analyses of reliability, validity, and potential common method biases. Section “Results” presents analytical findings and interpretations. The research concludes in Section “Discussion” with a synthesis of the results, acknowledgment of the study’s limitations, and suggestions for future research, ending with implications for industry professionals.

## Literature review and hypothesis development

### Green supply chain management (GSCM)

GSCM strategies have emerged in recent decades, widening their horizons by exploring their role in promoting green practices in supply chain processes to reduce negative environmental impacts, leading to sustainable growth^21^. It contributes to improved environmental performance^13^, increased profits, sales, and customer loyalty^22^. It helps improve environmental performance and provides an edge over others in intense competition in a globalized world^23^.

GSCM practices are based on numerous behavioral elements that have been widely researched in sector studies, such as in the mining industry^24^, the manufacturing sector^25,26^, and SMEs^27^. Researchers have identified driving factors that influence GSCM adoption, including characteristics inherent to the firm^28^ and factors intrinsic to supply chain members^29^. Under the firm’s inherent factors, strategic direction aids the implementation of GSCM practices^30^. Top managers’ role in adopting green practices is inevitable^31^, and senior management support significantly affects GSCM adoption^27^. Therefore, top management commitment is required for the smooth implementation of GSCM practices^32^, and total management interest is a crucial component of this process^33^. While peer pressure among supply chain members encourages the adoption of GSCM practices^34^, manufacturers are also under pressure because of the regulatory framework, investment recovery, CSR, and the green market^35^. This pursuit is also driven by a firm’s reputation strategy or public image^36^. Supply chain analytics provides actionable insights into managerial contributions by quantifying the impact of GSCM initiatives on performance metrics such as emissions reduction, procurement efficiency, and energy utilization. Analytics tools enable organizations to simulate scenarios, prioritize green investments, and optimize sustainability outcomes within their supply chains^37^.

However, manufacturers, specifically in developing nations, face difficulties encapsulating greener dimensions in every activity of the supply chain process. There is a scarcity of studies on industry-specific issues in developing countries and on measuring the social impact of supply chains^38^. While the literature on motivations and drivers for GSCM adoption and implementation is extensive, whether GSCM adoption leads to sustainable manufacturing and a sustainable society is debatable and understudied,therefore, this study’s contribution is multifaceted.

#### Green procurement and top management performance

Various decision-making tools have been developed to address the challenges faced while selecting appropriate suppliers^39^, as choosing the right supplier can help an organization adopt green practices^40^. Therefore, green procurement is considered an essential part of GSCM^41^, and a critical catalyst for implementing green practices^42^. It helps implement GSCM^12^ enables firms to conduct green manufacturing^43^. Green procurement has several advantages including customer satisfaction and increased market share^44^. Green procurement activities, such as supplier evaluation and collaboration for green practices, directly impact environmental performance^45^. Dubey et al.^44^ theorized a research framework to investigate how green procurement influences the top management performance of the Indian manufacturing sector. These activities improve financial and environmental performance^46^ and help firms gain competitive advantage over others^47^. Green procurement can serve as a means for firms to improve their environmental performance and top management can facilitate this process by promoting green innovation. Crucial green supply chain practices contribute to sustainability and reduce carbon emissions by selecting sustainable suppliers or outsourcing partners.^48^.

Green procurement has become an essential strategy for organizations to improve their environmental and social performance while enhancing their financial performance. Pagell et al.^49^ found that companies implementing green procurement practices reported higher profitability and return on assets than ROA.^50^ revealed that green procurement positively influences social responsibility and stakeholder management in the hospitality industry. Green procurement can positively impact top management performance in terms of financial, environmental, and social outcomes, contributing to long-term sustainable development.

Therefore, it is critical to examine the effects of “green procurement” on "top management performance," leading to the following hypotheses:

**H**_**1**_. Green Procurement significantly and positively impacts the Top Management Performance.

#### Green logistics and top management performance

Earlier researchers considered reverse logistics an essential part of GSCM^51^, as it encourages a bidirectional flow of goods^35^ and helps reduce costs^52^. However, green logistics has recently emerged as an essential concept for enhancing the long-term growth of logistics operations owing to the unification of the economy and society (Tan et al.,^53^). Green logistics are a foundational component that play an essential role in developing a Circular Economy by closing the loop^54^. It integrates environmental considerations into the logistics process^55^ by focusing on SCM solutions to reduce environmental footprint and handle waste while handling material, packaging, and transportation^54^. Partnerships with Suppliers for ecological sustainability will aid in ensuring the introduction of green logistics and further improving competitive advantage and management performance^56^. Green Logistics management positively affects environmental and operational performance^57^. Given its potential to reduce carbon emissions and promote environmental sustainability, green logistics have become crucial to modern supply chain management. Several studies explore the impact of green logistics on top management performance in terms of financial, ecological, and social outcomes. For instance,^58,62^, found that green logistics practices positively affected financial performance by reducing costs and enhancing customer satisfaction.

Furthermore, Hejazi et al.^59^ revealed that green logistics practices positively impact environmental sustainability, social responsibility, and corporate reputation. Burki et al. (2020) also demonstrate that green logistics practices improve customer loyalty, brand reputation, and overall business performance. The findings suggest that green logistics practices can positively impact top management performance in multiple dimensions, underscoring the importance of incorporating green logistics practices into supply chain management. Therefore, it is essential to capture the impact of “green logistics” on "top management performance," on top management performance and formulate the following hypothesis:

**H**_**2**_. Green Logistics significantly and positively impacts the Top Management Performance.

#### Regulatory framework and top management performance

Legislation is perceived as one of the primary reasons manufacturers feel tremendous pressure to improve their environmental performance^15^. Regulatory pressure on manufacturers influences their adoption of green purchasing^60^. However, the green regulatory framework should not be viewed as a barrier or restriction but as a tool for better risk management, revenue security, and credibility^61^. Companies in developing markets should be aware of their target markets’ local regulations^35^. Government support significantly impacts the implementation of green practices^62^. Suppliers can be encouraged to engage in green supply chain activities if the government supports them^63^— and financial policies related to loans and tax rebates can aid in implementing GSCM^35^. The relationship between green logistics and management performance improves because of regulatory pressure^57^. Various studies have explored the regulatory framework and its impact on top management. Boiral^64^ suggested that implementing environmental regulations can be facilitated by promoting organizational citizenship behaviors among employees. ^65^ examined the diversity of corporate governance in different countries and their impact on regulatory compliance. Javeed et al.^66^ found that environmental regulations positively affect firm performance. Chen et al.^67^ explored the impact of environmental regulations on corporate environmental performance in China and found a positive relationship. Delmas and Montes-Sancho^68^ examined the effectiveness of renewable energy policies implemented by U.S. states. The regulatory framework plays a crucial role in shaping top management’s actions towards environmental sustainability. Implementing effective regulations and policies can encourage organizations to adopt environmentally responsible practices^69^.

Therefore, to investigate the influence of the “regulatory framework” on "top management performance," the following hypothesis is proposed.

**H**_**3**_. Regulatory Framework significantly and positively impacts the Top Management Performance.

#### Green product & product design and top management performance

Green products comprise two types of components: new and recycled, guided by regulatory bodies^35^. Consumer environmental awareness (CEA) and reference behavior cause consumers to compare green products with conventional products in terms of price, functionality, and environmental friendliness^70^. Therefore, Eco-design or Green product design dramatically influences ecological, economic, cost-reduction, and intangible outcomes^52^. Government assistance through subsidies and refunds facilitates a smoother rollout of green products^35^. Many developed nations allow the use of green labels to differentiate between green products. It is not just compliance with regulations, but an opportunity to attract customers and gain a competitive advantage^70^.

The concept of Design for Environment (DfE) was founded and discussed by Allenby and Fullerton^71^, and Hosoda^72^ addressed the role of DfE in reducing the amount of end-of-life products (ELP). Green product design and innovation help businesses improve their productivity and core competencies^73^, competitiveness, dynamic capability^74^, and the success of green products. The overall performance of green product innovation improves because of its competitiveness and success^75^. Green product development performance is aided by a shared vision and organizational culture that promotes green practices, and environmental performance is boosted and improved by DfE^76^. Green products and product design have become essential strategies for organizations to improve their environmental and social performance while enhancing their financial performance. Several studies have explored the impacts of green products and product design on top management performance. A survey by Luchs et al.^77^ found that green product design can positively influence consumers’ perceptions of product quality, brand reputation, and willingness to pay premium prices.

Moreover, green product design can result in cost savings and improved efficiency, positively impacting financial performance. Green product design can enhance customer loyalty and brand reputation, and positively impact social performance. Green products and product design can positively influence environmental performance by reducing waste, energy consumption, and carbon emissions. Overall, green products and product design can positively affect top management performance in multiple dimensions, highlighting the importance of incorporating sustainability principles into product design and development.

Therefore, it is critical to comprehend the connection between "green product & product design" and "top management performance." Therefore, we formulated the following hypotheses:

**H**_**4**_. Green Product and product Design significantly and positively impact the Top Management Performance.

#### Top management performance and low carbon performance

GSCM activities significantly enhance corporate performance^28^ and improve economic performance^13^. The strategic orientation of an organization and its implementation are strongly associated^78,79^. Therefore, top management support is essential when adopting green practices^41^. Improved environmental and economic performance due to GSCM practices lead to positive operating efficiency and improved organizational performance^14^. While global environmental issues have led local manufacturers to adopt GSCM practices, they have also reported improved environmental and financial performance^80^. Furthermore, low-carbon supply chain incorporation enhances environmental and economic performance^81^. The key contributors to low carbon performance are environmental policies, political-legal regulations, and CSR activities supported by top management^82^. Shared responsibilities and cooperation among businesses can help manage the carbon footprint and reduce carbon emissions^41^. Hence, the following hypothesis was developed to investigate the linkage between "top management performance" and "low carbon performance."

**H**_**5**_. Top Management Performance significantly and positively impacts the Low Carbon Performance.

#### Low carbon performance and sustainable manufacturing & sustainable society

Monitoring ecological footprints is essential for minimizing the environmental damage caused by businesses^51^. The Green Supply Chain is a step forward in reducing the ecological footprint^51^. Sundarakani et al.^83^ were the first to examine the Carbon Footprint across the supply chain and proposed a model to measure it. Carbon emission costs are also vital as they may alter the overall configuration of the supply chain. Hence, while designing the supply chain, carbon emission costs should be carefully analyzed^84^. Blockchain technology helps to make supply chain operations efficient by monitoring carbon footprints and reducing carbon emissions^85^. The need for sustainable manufacturing has increased owing to conventional manufacturing, limited resources, and negative environmental impacts^86^. Manufacturing systems should be self-sustaining, wherein materials and residuals can be recycled and reused^21^. Applying GSCM practices contributes to sustainable performance^52^. One of the aims of a smart city is to promote a green and low-carbon footprint^41^. A sustainable society satisfies the current environmental, economic, and social needs without jeopardizing future needs. Responsible labor and green practices to reduce carbon footprints and streamline supply chain operations are essential for the efficient production of goods and services in a sustainable society^87^, (2018).

A growing body of research highlights the importance of top management commitment and involvement in low-carbon emission initiatives. Also, the predictive analytics models are particularly valuable for evaluating low-carbon performance. By integrating supply chain data such as carbon emissions and logistics metrics, organizations can simulate the effects of green practices and identify the most effective strategies for achieving sustainability goals (Massimiliano^9^.

For example, Yuan et al. (2015) found that top management support and commitment are critical determinants of the successful implementation of low-carbon practices in the supply chain. Similarly, Yijuan et al. (2020) found that top management support and involvement in carbon emissions reduction efforts are positively associated with improved firm performance. These studies suggest that top management is critical in driving low-carbon emissions initiatives and promoting sustainable practices within organizations^88^.

Therefore, it is critical to look into the effect of "low carbon performance" on “sustainable manufacturing” and on a "sustainable society," leading to the following hypotheses:

H6a: Low Carbon Performance significantly and positively impacts Sustainable Manufacturing.

H6b: Low Carbon Performance significantly and positively impacts the Sustainable Society.

### Theoretical model

**Fig. 1:**
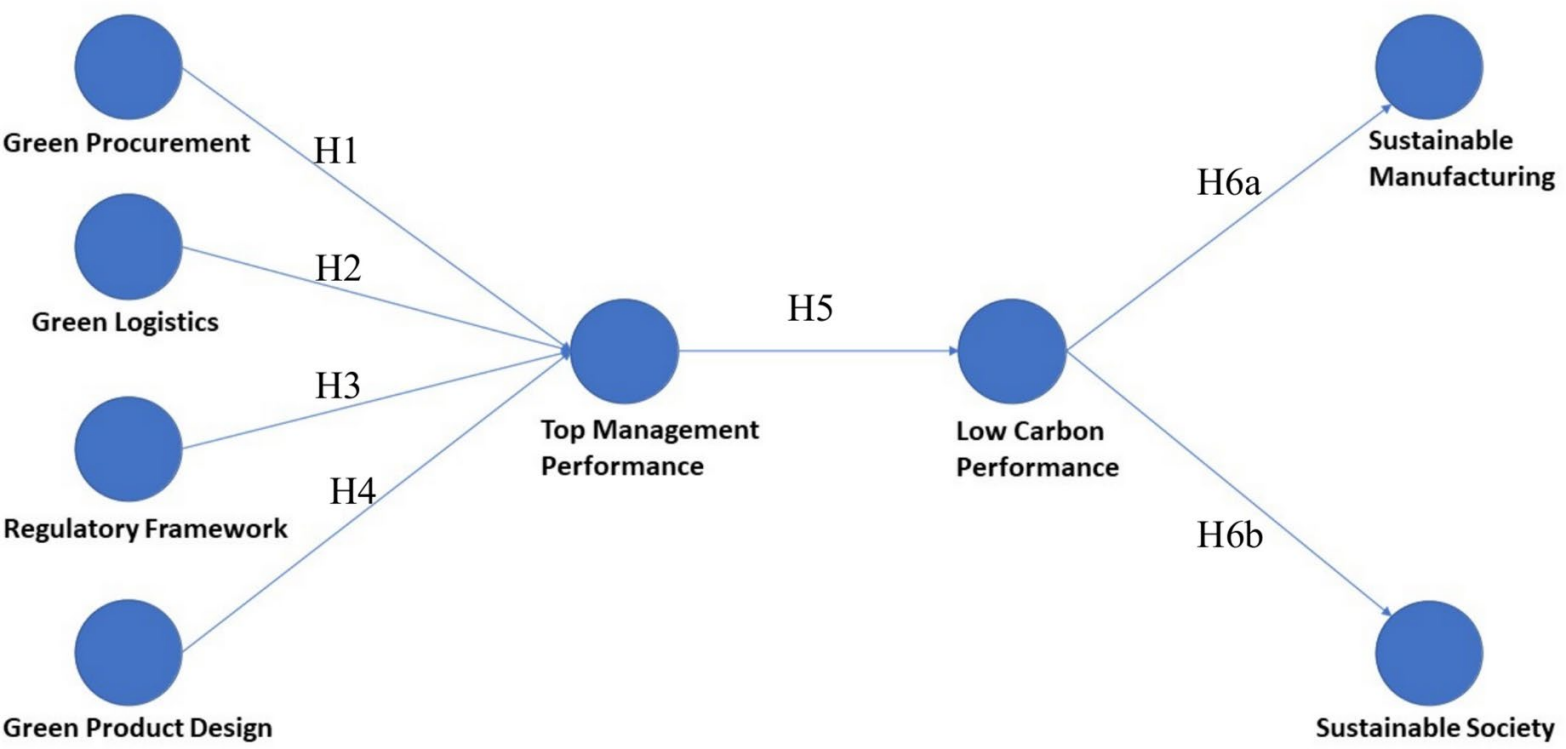
Conceptual model for green practices.

In Fig. 1, the hypotheses shown are theorized, and descriptions of the constructs defined in the model are discussed. The model on green practices includes constructs *"Green Procurement," "Green Logistics," "Green Product and Product Design," and “Regulatory Framework”* as antecedents and their relationship with the construct *"Top Management Performance."* The model further extends to explore the relationship between *"Top Management Performance"* with the construct *"Low Carbon Performance"* and the relationship between *"Low Carbon Performance"* with the constructs *“Sustainable Society” and “Sustainable Manufacturing”.* The eight constructs were measured by six, five, five, six, five, seven, five, and two indicator items, respectively.

## Methodology

The theoretical foundations of RBV and Stakeholder theory are complemented by empirical evidence derived from Structural Equation Modelling (SEM) to explore the relationship between GSCM practices and sustainable outcomes. The present study theorized and proposed a model for green practices, including well-defined constructs with clear hypotheses, robust data collection methods, and the use of SEM making it ideal to meet study’s objectives and to test relationships among constructs. This study employs a Structural Equation Modelling (SEM) approach to examine the relationships among Green Supply Chain Management Practices, Top Management Performance, and Sustainable Outcomes in Indian bag manufacturing enterprises. It advances previous works by emphasizing the role of top management performance as a mediating factor in achieving sustainable outcomes through GSCM. Data were collected through a structured survey distributed to top management personnel in various manufacturing firms. The data collected include a sample of top-, middle-, and junior-level managers working for bag manufacturing firms. Dubey et al.^44^ presented a model showing the role of green procurement in firm performance using a sample of the Indian manufacturing sector. However, the sample size was limited to 55 respondents, who focused only on green procurement. In contrast, the sample size of 389 is sufficient based on^89^ sampling formula, which deems this number adequate for a population exceeding 10,000 at a 95% confidence level. Additionally, the study incorporates constructs such as "green logistics," "green product and product design," and "regulatory framework.”

In “Sampling Techniques” (1977), Cochran provides a formula to determine sample size for large populations:

$${\text{n}}_{{\text{o}}} = {\text{Z}}^{2} *{\text{p}}*{\text{q}}/{\text{e}}^{2} \,{\text{where}}:$$

n₀ is the initial sample size, Z is the Z-value corresponding to the desired confidence level (e.g., 1.96 for 95% confidence), p is the estimated proportion of the population with the attribute of interest, q is 1 – p, e is the desired level of precision (margin of error).

For populations that are not large, Cochran suggests adjusting the sample size using the finite population correction:

$${\text{n}} = {\text{n}}_{{\text{o}}} /\left( {{1} + \left( {{\text{n}}_{{\text{o}}} - {1}} \right)/{\text{N}}} \right)$$

where N is the population size.

Applying these formulas: Initial Sample Size (n₀):

Desired confidence level: 95% (Z = 1.96).

Estimated proportion (p): 0.5 (assuming maximum variability).

Precision (e): 0.05.

$${\text{n}}_{{\text{o}}} = \left( {{1}.{96}} \right)^{2} *0.{5}*0.{5}/\left( {0.0{5}} \right)^{2} = {384}.{16}$$

Adjusted Sample Size (n):

Population size (N): 10,000

$${\text{n}} = {384}.{16}/\left( {{1} + \left( {{384}.{16} - {1}} \right)/{1}0,000} \right) \approx {37}0$$

A larger sample size improves the statistical inference of conclusions based on hypothesis testing. The sample was drawn using random and snowball sampling methods from a verified directory of Indian bag manufacturers. This approach ensures a representative demographic. Additionally, high response rates and tests for non-response bias further support the representativeness of the sample.

### Questionnaire design and data collection

The constructs for this study were derived from existing literature on Green Supply Chain Management and related fields. The compiled reports were validated with the help of experts to ensure a comprehensive evaluation of the validity and relevance of the questionnaire. A panel of experts comprised three experienced managers from the bag manufacturing industry who brought practical insights, two professors, and three research scholars researching the manufacturing industry, and offered a well-rounded academic perspective. This followed a pilot study that helped modify the questionnaire in terms of language and dropped a few statements based on poor reliability. The final questionnaire was used for data collection. The sample was drawn from a diverse range of Indian manufacturing firms, including large and small bag manufacturing firms, by referring to the online directory of bag manufacturers on the Exporters India. The study ensured that respondents were from firms actively implementing GSCM practices. Data integrity was further strengthened by cross-verifying responses with secondary sources like sustainability reports and environmental certifications.

Ethical approval was not required for this study as it did not involve procedures necessitating institutional review board approval. However, informed consent was obtained from all participants prior to their inclusion in the study. The study was approved by the Institutional Advisory Committee of the Atal Bihari Vajpayee School of Management and Entrepreneurship, Jawaharlal Nehru University, New Delhi, India. Participants were informed of the study’s objectives, the voluntary nature of their participation, and their rights, with consent obtained prior to their involvement in the study. The questionnaire was created using Google Forms, and its link was emailed to managers working with selected firms adopting GSCM practices as per the given criterion in Table 1.

**Table 1:** Standards for selection of firms.

Firm type	Environmental certifications	Sustainability reports	Green procurement verification	Green logistics verification
Bag Manufacturing Firms	ISO 14,001, FSC Certification, BIS Eco-Mark (ISO, 2025; FSC, 2022; BIS, 2023)	Extended Producer Responsibility (EPR) filings, CII Green Co ratings, IGBC reports	Supplier records cross-checked with regulatory compliance reports	Real-time tracking systems, route optimization software, carbon footprint monitoring

A total of 480 responses were received in six months (March 2020 to Sep 2020). Responses that did not meet the requirements for implementing GSCM practices in their manufacturing processes were eliminated during the data-gathering procedure. To preserve the integrity of the dataset and concentrate on manufacturers committed to sustainability and green efforts, the response elimination criteria were set to include only those who had implemented GSCM practices in their business operations, leading to a total sample of 389. This meticulous methodology guaranteed that the gathered data accurately represented the intended demographics of interest. This study used a 5-point Likert scale ranging from “Strongly Disagree” to “Strongly Agree”. The 5-point Likert scale was consistent with the approach used in previous studies to measure these constructs for the responses, and no missing data were allowed in the Google forms by making all fields mandatory to be filled. The link was also sent to top managers using personal networks, and in this manner, the snowball sampling method was also used in the study. First, interactions with bag producers were recognized for their environmentally friendly and sustainable supply chain procedures. Next, we asked these first contacts to recommend more firms that fit the exact requirements, using the snowball process. We kept going through This iterative process continued until saturation was reached, which guaranteed a broad and representative sample of manufacturers dedicated to green supply chain practices. Non-response bias was also examined by comparing the early and late respondents using an independent sample t-test.

### Sample demographics

The demographic variables of the survey respondents are presented in Table 2.

**Table 2:** Demographic profiles of the participants.

Demographics	Subcategories	Frequency (Percentage)
Gender:	Female	170 (43.70%)
	Male	219 (55.01)
Designation	Junior managers	166 (42.67%)
	Middle-level managers	135 (34.70%)
	Top managers	98 (25.19%)
Age group	Age between 25 and 35	139 (35.73%)
	35–50	104 (26.73%)
	Above 50	146 (37.53%)
Work experience	Less than ten years	182 (46.78%)
	Between 10 and 20 years	109 (28.02%)
	Above 20 years	98 (25.19%)

Table 1 represents the demographic profiles of 389 respondents, which 56.29% of the participants are male and 43.70% were female. 42.67% Of the respondents are junior managers, 34.70% were middle-level managers, and 25.19% were from top management of the firms. The sample consisted of 35.73% of participants in the age group 25–35 years, followed by the participants in the age group 35–50 years (26.73%), and the remaining 37.53% were in the age group above 55 years, respectively. The above table shows that 46.78% of the participants have an experience of fewer than 10 years, and 28.02% understand 10–20 years. 25.19% of the participants have more of above 20 years, experience. The sample size of 389 is considered representative, and the conclusions made in this study can be generalized for several reasons. First, the sample size is sufficient to represent the entire population. India has over 10,000 bag producers^90^. A sample size of 389 is adequate to represent the population, representing around 4% of the total population (Cochran,^89^). Second, a database of Indian bag manufacturers randomly selected the sample. This guarantees that the sample, rather than the small number of businesses, is representative of the entire Indian bag manufacturing sector. Third, more than 70% of respondents completed the survey. This means that the sample is likely to represent the bag manufacturing industry in India as it is not biased towards companies that are more likely to respond to surveys.

### Structural equation modelling

The primary response data were analyzed using partial least squares structured equation modeling (PLS-SEM). Structural equation modeling (SEM) evaluates and validates intricate hypotheses and models^91^. It is an effective technique that can be used to examine interactions directly or indirectly between various variables^92^. SEM has been applied in several domains such as marketing, psychology, business, and education^93^. Recently, GSCM was studied using SEM. GSCM is a relatively new area for assessing the effects of supply chain management on society and the environment^94^. SEM may shed light on the connections between elements that affect GSCM performance in an intricate and dynamic environment. Before analyzing the interrelationships among the constructs, it is essential to analyze the measurement model for construct validity and reliability^95,96^. Therefore, the model was examined for internal consistency, reliability, and convergent and discriminant validity on a scale. The results of testing the hypothesized measurement model using the PLS algorithm are reported in the subsequent subsections. The structural equation modeling (SEM) approach adopted in this study aligns with supply chain analytics by quantifying the relationships among constructs such as green procurement and low-carbon performance. These relationships can be extended into predictive models to forecast the impact of green practices on sustainability outcomes, providing actionable insights for supply chain decision-makers.

### Reliability and validity analysis

In this study, Cronbach’s alpha was calculated to investigate internal consistency reliability^97^, which measures the pairwise correlation between items and determines the extent to which a group of items or statements on a scale is homogeneous. Its value ranges between zero and one,however, the expected value is greater than 0.7. The construct validity of the measurement model was examined using confirmatory factor analysis (CFA) method. Construct validity includes convergent and discriminant validity. Convergent validity was examined using construct loadings, composite reliability, and average variance extracted variance. Discriminant validity was reviewed using the Fornell–Larcker criteria. Table 3 presents the results of the reliability and validity analyses.

**Table 3:** Illustrates the psychometric properties of the measurement model in terms of reliability, convergent validity, and discriminant validity.

Construct	Items	Description	Standard factor loading	Cronbach alpha	Composite reliability	Average variance extracted
Green procurement	GP1		0.681	0.873	0.874	0.531
	GP2		0.767			
	GP3		0.753			
	GP4		0.761			
	GP5		0.743			
	GP6		0.682			
Green logistics	GL1		0.604	0.836	0.832	0.502
	GL2		0.721			
	GL3		0.781			
	GL4		0.672			
	GL5		0.779			
Green Product and Product Design	GD1		0.819	0.896	0.898	0.633
	GD2		0.798			
	GD3		0.780			
	GD4		0.804			
	GD5		0.780			
Regulatory Framework	RF1		0.712	0.871	0.871	0.531
	RF2		0.771			
	RF3		0.748			
	RF4		0.698			
	RF5		0.767			
	RF6		0.673			
Top Management Performance	TMC1		0.762	0.889	0.887	0.613
	TMC2		0.740			
	TMC3		0.736			
	TMC4		0.844			
	TMC5		0.836			
Low Carbon Performance	CP1		0.663	0.882	0.874	0.505
	CP2		0.774			
	CP3		0.731			
	CP4		0.774			
	CP5		0.786			
	CP6		0.695			
	CP7		0.622			
Sustainable Society	SS1		0.738	0.894	0.892	0.582
	SS2		0.790			
	SS3		0.776			
	SS4		0.792			
	SS5		0.751			
Sustainable Manufacturing	SM1		0.682	0.729	0.730	0.576
	SM2		0.841			

A Cronbach’s alpha value exceeding 0.7 is deemed “acceptable” for internal consistency, while a value of 0.8 or higher is categorized as “good”^98^. As shown in Table 2, Cronbach’s alpha for the eight constructs, ranging from 0.729 to 0.896, has acceptable and good internal consistency reliability. The included measures were found to be reliable, valid, and suitable for studies using SEM analysis. Composite reliability (C.R.) checks construct reliability and convergent validity in a measurement model. It assesses the construct’s consistency, as well as the construct’s stability and equivalence, and offers a more retrospective means to overall reliability^92^. The existence of adequate scale validity can be ascertained by a C.R. value larger than 0.7^95,99^.

The composite reliability values in Table 3, ranging from 0.730 to 0.898, show that the eight constructs had good reliability. Convergent validity is the degree to which construct items converge or share a large amount of their variance^92^. Convergent validity was assessed through standardized construct loadings. For a construct to be deemed valid, its standardized loadings should exceed 0.50^92^. Table 2 shows that the range of construct loadings for the observed variables was between 0.604 and 0.844. This range indicates that the observed items reliably and precisely represented their respective constructs. Therefore, we are confident in the presence of convergent validity.

Furthermore, the scale’s discriminant validity shows how a construct is unrelated to others^92^. When analyzing discriminant validity, the average variance extracted (AVE) estimates for every construct must be above the highest shared variances of the individual constructs, and the square root of AVE must be above the correlation between constructs. Table 4 demonstrates that each construct has a weak but positive correlation with the others, implying that they are all independent constructs. Furthermore, in all of the constructs (bold values in Table 4), the square roots of AVE were higher than the non-diagonal associated values. These findings explain the strong correlation of each construct with its items.

**Table 4:** Correlation matrix and roots of AVE’s.

	Sustainable. society	Green procurement	Green logistics	Green product design	Regulatory framework	Top. management performance	Low carbon performance	Sustainable manufacturing
Sustainable Society	**0.770**							
Green Procurement	0.367	**0.732**						
Green logistics	0.342	0.242	**0.715**					
Green product design	0.373	0.367	0.444	**0.796**				
Regulatory framework	0.198	0.208	0.330	0.295	**0.729**			
Top management performance	0.427	0.307	0.388	0.456	0.265	**0.785**		
Low carbon performance	0.378	0.332	0.340	0.443	0.251	0.403	**0.723**	
Sustainable manufacturing	0.378	0.256	0.344	0.373	0.213	0.648	0.335	**0.766**

*The diagonal in bold represents the square root of average variance extracted from observed variables (items); Off-diagonal represents correlations between constructs.

*The diagonal values in bold represent the square root of AVE from observed variables (items), whereas off-diagonal represent correlations between constructs.

The HTMT ratio was used to test the discriminant validity of the measurement model. Table 5 reports the results of the HTMT ratio, where the estimated ratio for each pair of constructs was less than the required value of 0.8. Thus, the results support the discriminant validity of the measurement scale.

**Table 5:** Heterotrait-monotrait ratio (HTMT).

	Green Logistics	Green Procurement	Green Product Design	Low Carbon Performance	Regulatory Framework	Sustainable Manufacturing	*Sustainable Society*
Green Logistics							
Green Procurement	0.259						
Green Product Design	0.451	0.369					
Low Carbon Performance	0.347	0.336	0.451				
Regulatory Framework	0.330	0.209	0.294	0.252			
Sustainable Manufacturing	0.362	0.279	0.398	0.348	0.233		
Sustainable Society	0.352	0.371	0.373	0.381	0.192	0.396	
Top Management Performance	0.414	0.317	0.470	0.416	0.283	0.629	0.436

### Common method bias (CMB)

CMB was also examined and reported after validating the internal consistency reliability and construct validity of the measurement scale to address potential biases that details how self-reporting bias was minimized. (i) We conducted Harman’s single-factor test, which confirmed that no single factor explained more than 26.48% of the variance, ensuring that common method bias is unlikely to distort findings. According to the obtained values, the variance explained by a single factor was 26.48%, smaller than 50%^100^. Thus, it can be concluded that the responses were clear from common method bias, and all the conclusions made in the study were free from bias (Table 6). (ii) We cross-verified self-reported data with sustainability reports and environmental certifications where available to enhance credibility (Table 1).

**Table 6:** Common method bias.

Total variance explained						
Component	Initial eigenvalues			Extraction sums of squared loadings		
	Total	% of Variance	Cumulative %	Total	% of Variance	Cumulative %
1	11.389	26.485	26.485	11.389	26.485	26.485
2	3.348	7.785	34.271			
3	3.153	7.332	41.603			
4	2.815	6.546	48.149			
5	2.472	5.750	53.899			
6	2.181	5.071	58.970			
7	1.965	4.570	63.539			
8	1.067	2.481	66.020			
9	0.770	1.792	67.811			
10	0.765	1.779	69.591			
11	0.671	1.561	71.152			
12	0.638	1.485	72.636			
13	0.620	1.442	74.079			
14	0.573	1.333	75.412			
15	0.564	1.312	76.724			
16	0.530	1.233	77.957			
17	0.516	1.199	79.156			
18	0.512	1.190	80.347			
19	0.494	1.148	81.495			
20	0.488	1.135	82.630			
21	0.466	1.085	83.715			
22	0.461	1.073	84.788			
23	0.456	1.061	85.848			
24	0.436	1.014	86.863			
25	0.417	0.969	87.832			
26	0.396	0.922	88.754			
27	0.388	0.902	89.656			
28	0.387	0.900	90.556			
29	0.379	0.882	91.438			
30	0.365	0.848	92.286			
31	0.344	0.799	93.086			
32	0.338	0.785	93.871			
33	0.323	0.751	94.622			
34	0.313	0.728	95.350			
35	0.306	0.711	96.061			
36	0.302	0.701	96.763			
37	0.278	0.646	97.408			
38	0.264	0.614	98.023			
39	0.252	0.585	98.608			
40	0.232	0.541	99.149			
41	0.209	0.487	99.636			
42	0.157	0.364	100.000			
43	-2.963E-15	-6.891E-15	100.000			

Extraction Method: Principal Component Analysis

## Results

### Structural model and hypothesis testing

**Fig. 2:**
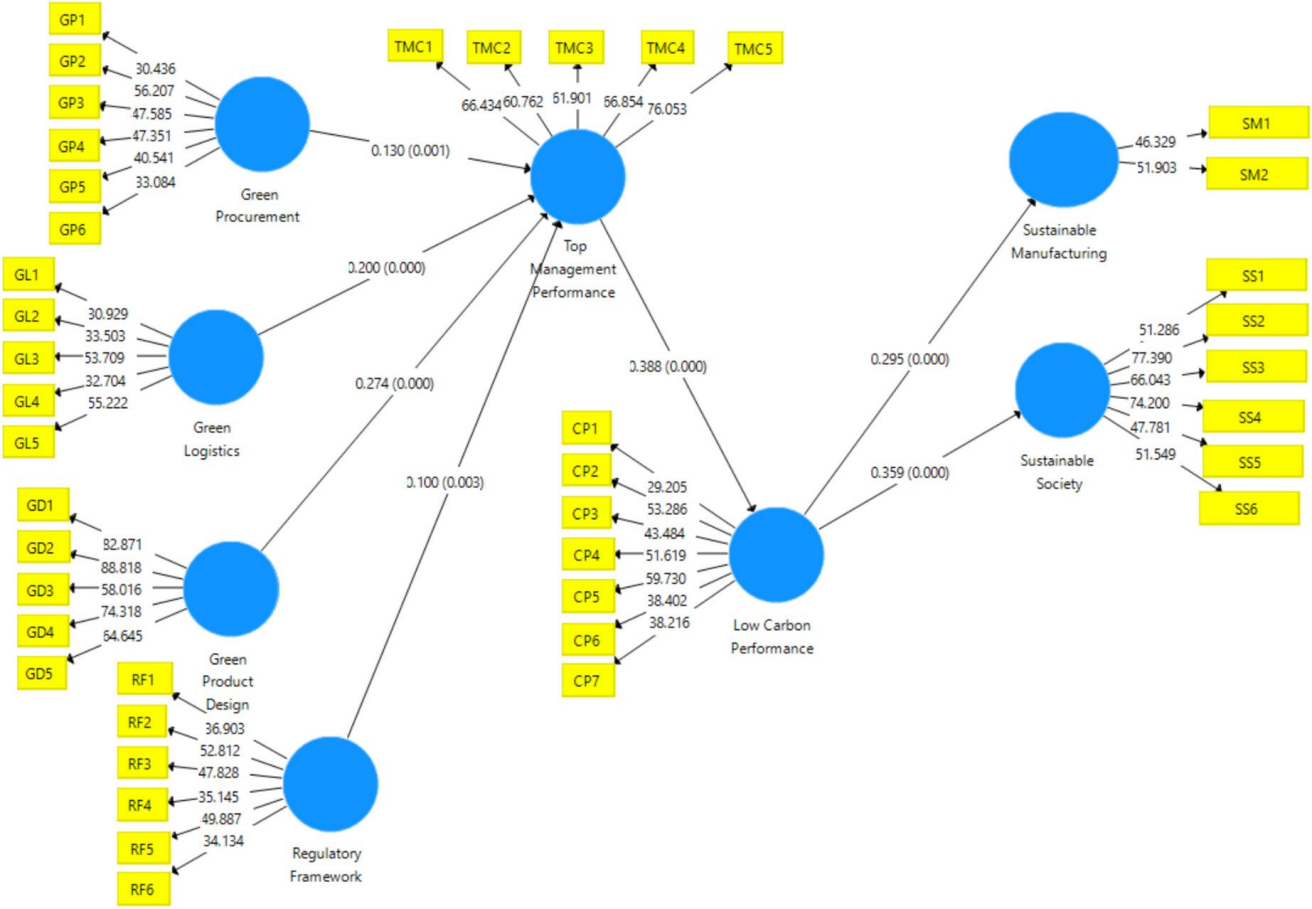
Structural model representing the significant relationships.

SEM in SMART PLS software was used to evaluate the hypothesized conceptual research model. The structural model is shown in Fig. 2, and Table 7 summarizes the findings of the SEM analysis.

**Table 7:** Results of hypothesis testing using SEM analysis.

Endogenous Construct	Exogenous Construct	Standardized Estimate	Standard Error	*p* values	R Square	Remark
Top Management Performance	Green Procurement	0.130	0.038	0.000	20.7%	Supported
Top Management Performance	Green Logistics	0.200	0.046	0.000		Supported
Top Management Performance	Green Product and Product Design	0.274	0.044	0.000		Supported
Top Management Performance	Regulatory Framework	0.100	0.037	0.007		Supported
Low Carbon Performance	Top Management Performance	0.388	0.036	0.000	19.5%	Supported
Sustainable Society	Low Carbon Performance	0.359	0.032	0.000	16.1%	Supported
Sustainable Manufacturing	Low Carbon Performance	0.295	0.037	0.000	14.7%	Supported

Table 7 explains the estimated values of the endogenous constructs of standardized path coefficients (β), standard error, t-statistics, *p*-value, and R-square. The significance level (α) was set at *p* < 0.05. The hypothesis testing results are shown in Table 7, with each beta coefficient explaining the relative value of the influencing factor. All path coefficients were positive. The four elements chosen, namely "green procurement,” green logistics, “regulatory framework," and "green product and product design," all have a significant impact on "top management performance." The most important factor influencing the "top management performance" is "green product and product design," compared to the remaining elements (path coefficient = 0.274, *p*-value = 0.000), emphasizing the role of product innovation in sustainable manufacturing. Furthermore, the firm’s " low-carbon performance" was found to be significantly and positively influenced by "top management performance (path coefficient = 0.388, *p*-value = 0.000), reinforcing the importance of leadership commitment in sustainability. The firm’s "low carbon performance" was discovered to impact the "sustainable manufacturing significantly" (path coefficient = 0.295, *p*-value = 0.000). The moderate effect size indicates that firms reducing carbon footprints also improve their sustainable production methods. The result also supported the hypothesis that the "low carbon performance" of the firms positively influences the “sustainable society” (path coefficient = 0.359, *p*-value = 0.000). Reductions in carbon emissions lead to broader societal benefits, such as improved environmental quality and corporate social responsibility outcomes. The estimations align with expectations, because the association is significant (< 0.05) and in the anticipated direction. Green product & product design (β = 0.274, *P* < 0.001) highlighting that firms focusing on sustainable product innovation experience the highest management efficiency improvements. The regulatory framework has a positive and statistically significant impact on top management performance, but the effect size is the smallest among all predictors (β = 0.100). This suggests that compliance with environmental regulations supports management decisions, though it is not the strongest driver.

The strongest predictor of Top Management Performance was Green Product & Product Design. The most influential factor on Low Carbon Performance was Top Management Performance.

The transition from Low Carbon Performance to Sustainable Manufacturing and Sustainable Society confirms the broader impact of corporate sustainability initiatives. This suggests that compliance with environmental regulations supports management decisions, though it is not the strongest driver.

The R-squared value demonstrates the robustness of the model. The model, as a whole, explained 20% of the "top management performance," 19.5% of the variations in "low carbon performance," 16.1% of "sustainable society," and 14.7 percent of variations in "sustainable manufacturing."

### Predictive relevance and external validity

Table 8 reports the f-square statistics, which are a standardized measure of the effect size. The f-square explains how much change in R^2^ accounts for a significant proportion of the unexplained variance^101^. The f square effect size value equal to and above 0.02 is considered as "small," a value equal to and above 0.15 is regarded as "medium," and a value equal to and above 0.35 is considered as “high” effect size (Cohen,^89,102^). Table 8 explains that "Top Management Performance" to "Low Carbon Performance" has a “medium” effect size (0.215), "Low Carbon Performance" to “Sustainable Manufacturing” has a “small” effect size (0.139), and "Low Carbon Performance" to “Sustainable Society” has a “medium” effect size (0.176).

**Table 8:** F Square statistics.

	Low carbon performance	Sustainable manufacturing	Sustainable society	Top management performance
Green Logistics				0.053
Green Procurement				0.022
Green Product Design				0.087
Low Carbon Performance		0.139	0.176	
Regulatory Framework				0.012
Sustainable Manufacturing				
Sustainable Society				
Top Management Performance	0.215			

Table 9 illustrates the construct cross-validated redundancy that determines the constructs’ predictive relevance^101^. In the SEM model, the Q square statistic, represented as 1 – (SSE/SSO) squared prediction error divided by squared observations, measures predictive relevance. By applying blindfolding algorithms, it seeks the model’s ability to predict the endogenous construct^103^ by repeating the observed values^104^. The Q squared values provide an insightful quantitative evaluation of the model’s prediction ability concerning many critical endogenous constructs. These values are essential tools for comprehending how well the model predicts the behavior and results of these constructs. The Q square value equal to and above 0.02 means "small," a value equal to and above 0.15 means "medium," and a value equal to and above 0.35 means “high” predictive relevance for the endogenous constructs (Cohen,^89,102^). Table 8 shows that the Q square values for the various constructs are as follows: Top Management Performance (0.237), Sustainable Manufacturing (0.040), low-carbon performance (0.152), and Sustainable Society (0.054), which often fall into the category of “moderate” predictive significance, implying that the model has moderate predictive power for various constructs. With a “medium” degree of predictive relevance, Top Management Performance stands out as particularly good, suggesting a relatively greater capacity for model forecasting.

**Table 9:** Q square estimates.

	Q^2^ predict	RMSE	MAE
Low Carbon Performance	0.152	0.923	0.780
Sustainable Society	0.054	0.976	0.880
Sustainable Manufacturing	0.040	0.983	0.823
Top Management Performance	0.237	0.876	0.683

Table 10 represents the prediction statistics, showing the CVPAT, where the structural model is compared with the linear regression model. The reported values represent the difference between average loss difference (Linear model minus PLS model). A negative and average loss difference suggests that the predictive power of the structural model is better as compared to the linear model. Thus, it can be inferred that the PLS model outperformed the linear model in terms of its predictive accuracy.

**Table 10:** Prediction statistics CVPAT.

	PLS loss	IA loss	Average loss difference	t value	p value
Low carbon performance	0.994	1.088	− 0.094	10.634	0.000
Sustainable society	1.198	1.242	− 0.044	9.987	0.000
Sustainable manufacturing	0.962	0.993	− 0.031	8.983	0.000
Top management performance	0.884	1.054	− 0.171	7.035	0.000
Overall	1.024	1.116	− 0.092	10.726	0.000

**Fig. 3:**
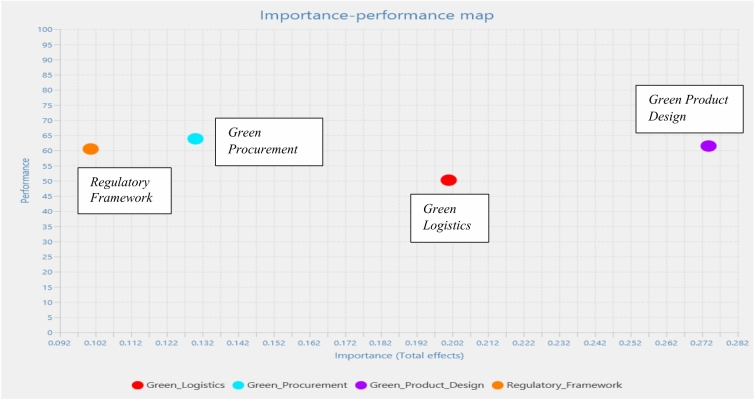
Importance and performance analysis (IPMA) results.

Figure 3 shows how Importance-Performance Map Analysis (IPMA) determines the relative significance of latent variables within the PLS model using path information. While importance represents the absolute total effect on the final endogenous variable, performance reflects the size of latent variable scores. Importance was measured on the x-axis. “Green Logistics” has higher absolute importance than the other constructs. The performance was calculated on the y axis. “Green Procurement” reflects higher mean latent scores than the other constructs, which means it has more robust measurement paths.

The SEM findings reveal significant relationships between green practices and sustainability outcomes. Supply chain analytics can operationalize these results through predictive models. For instance, the relationship between top management performance and low-carbon performance can be quantified using models such as:

This model predicts the expected improvements in low-carbon performance based on varying levels of top management support, allowing organizations to prioritize interventions. Similarly, the relationships between low-carbon performance and sustainable outcomes (e.g., manufacturing and society) can be modeled to provide precise forecasts for sustainability improvements (See Table 11).

**Table 11:** Predictive Models Linking GSCM Constructs with Supply Chain Analytics Applications.

Predictive model	Inputs	Output	Application
LCP = 0.375 * TMP	Top Management Performance (TMP)	Low-Carbon Performance (LCP)	This equation quantifies the influence of top management performance on low-carbon performance, allowing firms to estimate the expected carbon reductions driven by managerial decisions
SM = 0.283 * LCP	Low-Carbon Performance (LCP)	Sustainable Manufacturing (SM)	This predictive model enables organizations to project improvements in manufacturing sustainability for incremental gains in low-carbon performance
SS = 0.347 * LCP	Low-Carbon Performance (LCP)	Sustainable Society (SS)	This model highlights the societal benefits achievable through enhanced carbon performance, aiding policymakers in understanding broader sustainability impacts
TMP = 0.13 * GP + 0.20 * GL + 0.27 * GPD	Green Procurement (GP), Green Logistics (GL), Green Product Design (GPD)	Top Management Performance (TMP)	This demonstrates the combined influence of green practices on managerial performance
Conceptual: GP = f(SP, SC, CR)	Supplier Performance (SP), Supplier Collaboration (SC), Cost Reduction (CR)	Green Procurement (GP)	This equation guides procurement decisions by integrating supplier metrics and cost data
Conceptual: CO2 = f(LCP, GL)	Low-Carbon Performance (LCP), Green Logistics (GL)	Carbon Emissions (CO2)	Finally, it simulates the effect of logistics efficiency and carbon initiatives on emissions reductions

Supply chain analytics enhances the practical application of the findings by offering predictive and prescriptive insights. These predictive models illustrate how the constructs explored in this study can be operationalized using supply chain analytics. For example, the equation TMP = 0.13 * GP + 0.20 * GL + 0.27 * GPD provides a framework for assessing the impact of green practices on managerial effectiveness. Using supply chain analytics tools, firms can simulate how changes in green procurement, logistics, or product design translate into improved performance metrics.

Similarly, conceptual models such as GP = f(SP, SC, CR) and CO2 = f(LCP, GL) highlight the potential of supply chain analytics in optimizing procurement and emissions management. By integrating supplier performance and collaboration metrics into green procurement strategies, firms can make informed decisions that balance cost efficiency with sustainability goals.

## Discussion

The model for green practices is theorized and evaluated, comprising of "green procurement," "green logistics," "green product & product design," and "regulatory framework," and study the influence of these factors on "top management performance." The hypotheses examining the impact of these factors on "top management performance" were supported. It was found that the elements "green procurement," "green logistics," "green product & product design," and "regulatory framework significantly and positively influence the "top management performance." Global leaders focus on environmental conservation, and companies worldwide are taking the initiative to adopt green practices that different stakeholders appreciate. Our model substantiates the results of past studies investigating the effect of "top management performance" because of “green procurement”^44,46,47,105^, “green logistics”^56,57^, "green product & product design" (Nuryakin and Maryati,^70,73–76^ and “regulatory framework”^57^. Among the four factors, green products and product design are the most influential factors affecting top management performance. The model further probes how "top management performance" influences "low carbon performance." influences low-carbon performance. The hypothesis is supported, and evidence shows that "top management performance" significantly and positively influences "low carbon performance." Adopting green practices and their positive impact on top management performance also lead to low carbon performance, which is further rewarded in different ways. This model corresponds to earlier findings on the influence of "top management performance" on "low carbon performance"^41,81,82^. Further, the hypotheses to understand the effect of "low carbon performance" on “sustainable manufacturing” and on the “sustainable society” were investigated. The "low carbon performance" by the firms significantly and positively influences “sustainable manufacturing” as well as "sustainable society." Low carbon performance, which is the utmost requirement to preserve the environment, contributes significantly towards a sustainable society and manufacturing. This study establishes a link between green practices and the purpose of sustainable manufacturing and a sustainable society for everyone to survive.

This alignment, has a distinct focus from Schmidt et al.^17^, and emphasizes the novelty of our approach in examining the intricate dynamics between top management performance and green supply chain management. Our research contributes to the literature by providing nuanced insights into how top management’s commitment and performance can significantly shape the adoption and effectiveness of green practices within the supply chain. Our findings have significant implications for green supply chain management (GSCM) practices, particularly regarding how top management’s commitment and strategic alignment influence the adoption and effectiveness of these practices.

However, it provides additional insights specific to India, such as the role of regulatory frameworks and cultural emphasis on hierarchical management, which differs from decentralized management systems observed in Western contexts.

In line with^16^, we meticulously considered the potential impact of common method bias in our analysis, thereby strengthening the reliability of our conclusions. Our study underscores the critical role of leadership in steering GSCM initiatives, aligning with global sustainability goals, and fostering a culture of environmental responsibility within organizations.

## Conclusion

The present study contributes novel theoretical insights to the existing literature on green supply chain management by illuminating the nuanced interplay between top management performance as a mediating factor and sustainable supply chain initiatives. While previous research, as discussed by Schmidt et al.^17^, has explored the implications of green practices for firm performance, our study extends this dialogue by examining the specific mechanisms through which top management influences these outcomes. This approach not only addresses the critique of the lack of novelty but also enriches the theoretical tapestry by embedding GSCM within a broader strategic management context. This study highlights the importance of integrating supply chain analytics into GSCM practices. By applying predictive models to the relationships identified in this study, organizations can forecast and optimize sustainability outcomes. Supply chain analytics thus acts as a critical enabler, transforming theoretical insights into actionable strategies for achieving operational and environmental goals.

### Limitations and future research

This study uses a comprehensive approach that goes beyond adopting standard green practices to improve a company’s performance. It further explores how “sustainable manufacturing” and “sustainable society” are influenced. Consequently, the model’s formulation included a range of constructs. Separate studies on each construct can provide deeper insights. The gaps in the data verification process were addressed through targeted mitigation strategies. One major limitation was the lack of industry-wide sustainability data, as small and medium enterprises (SMEs) often do not publish sustainability reports, making verification challenging. This issue was mitigated by cross-referencing data against BIS Eco-Mark certifications, Extended Producer Responsibility (EPR) filings, and compliance records from State Pollution Control Boards (SPCB). Another challenge was the variability in environmental compliance, as regional enforcement of regulations such as ISO 14,001 and Forest Stewardship Council (FSC) standards was inconsistent. To address this, data was validated using CII Green Co ratings and reports from the Indian Green Building Council (IGBC) to ensure adherence. Additionally, supplier claims on green procurement posed a risk, as firms might overstate their use of biodegradable or recycled materials. To counter this, sourcing claims were verified through EPR compliance for plastics and FSC certification for jute and paper bags. Potential biases in data collection, particularly from self-reported company data, were carefully accounted for through specific mitigation strategies. One significant concern was self-reporting bias, where companies might overstate their green procurement practices, such as making false claims about biodegradable materials. To address this, supplier records and regulatory compliance reports were cross-checked to verify authenticity. Another issue was the over-representation of large firms, as small and medium enterprises (SMEs) often lack structured reporting, which could skew the results. This was mitigated by ensuring a diverse sample that included corporates, SMEs, and cottage industries. Additionally, limited awareness among small manufacturers posed a challenge, as some firms lacked knowledge of sustainability metrics. To improve data accuracy, guidelines on green supply chain metrics were provided to these firms, enhancing their understanding and reporting practices. Researchers have discovered the role of blockchain in smooth green logistics operations (Tan et al., 2020) and the tracking of carbon footprints in a supply chain using blockchain^85^. Using this model, they further explored the impact of blockchain use at various stages of green practices. However, the study’s limitations include a focus on the bag manufacturing industry, limiting cross-sector generalizability, the use of cross-sectional data restricting analysis of temporal effects and causality, and potential cultural and geographical biases from an exclusively Indian sample.

Future research should collect data from multiple informants across different organizational levels and functions to enhance the robustness of the findings. Using this model, further comparative research can be conducted among countries based on development, management support, and consumer sensitivity towards green manufacturing. Additionally, longitudinal studies can provide deeper insights into the evolving dynamics of green supply chain practices and managerial performance. Expanding the research scope to include diverse industries and geographical locations would also enrich our understanding of GSCM’s global applicability and effectiveness of GSCM.

### Implications for practitioners

This model presents an overview of how green practices contribute to sustainable manufacturing and society. Both these aspects are essential from a managerial perspective. Sustainable manufacturing ensures that the manufacturing unit follows economically viable policies and procedures, and processes that ensure low consumption of natural resources and energy have a minimum adverse impact on the environment. This is a joint effort that requires support from all supply chain stakeholders. While various studies prove that green practices contribute to top management performance, it cannot be denied that such practices can also positively contribute to long-term sustainable manufacturing. Therefore, the long-term goals of manufacturing organizations should focus on achieving sustainable manufacturing along with top management performance. Similarly, these organizations are also responsible for society around them, particularly corporate social responsibility (CSR). A sustainable society revolves around protecting the planet by ensuring a safe and secure environment to make it liveable for future generations. As manufacturers consume a lot of natural resources directly or indirectly, they are responsible for paying back to society by contributing towards a healthy ecosystem, becoming recycle-oriented, and ensuring low-carbon performance. Both a sustainable society and sustainable manufacturing are the long-term results of adopting green practices. Therefore, framing policies and adopting advanced technologies in the manufacturing and supply chain processes should be considered.

The link between " low-carbon performance and "Top Management Performance" has a medium effect size, indicating a strong and discernible correlation between the two variables. When senior management actively supports and implements these measures, an organization will likely make significant strides toward sustainability and carbon emissions reduction. Therefore, organizations need to understand the importance of top management backing and strong leadership to drive their low-carbon and sustainability programs effectively. This may entail establishing precise objectives, assigning funds, and promoting eco-friendly procedures across companies. Similarly, the association between " low-carbon performance and “Sustainable Manufacturing” has a negligible effect size, suggesting that there is less of a relationship between these two variables. From a practical standpoint, this implies that while attaining low-carbon performance is one aspect that impacts sustainability within the manufacturing process, it contributes to sustainable manufacturing. Beyond reducing carbon emissions, organizations seeking to improve sustainable manufacturing must take a more comprehensive approach to practices and tactics. This may entail trying to reduce waste, maximize the use of resources, and guarantee ethical sourcing and production procedures. The small effect size emphasizes the need for a comprehensive strategy for sustainable production. The relationship between "Low Carbon Performance" and “Sustainable Society” shows a medium effect size, suggesting that low carbon performance has a slight influence on promoting sustainability in society. This indicates that organizations may support larger societal sustainability goals by actively working to lower their carbon footprints and encourage sustainable practices. Practically speaking, companies may positively impact their communities and society by enacting environmentally conscious policies and participating in socially conscious activities. This might include assisting with neighborhood environmental projects, giving charitable causes, or lessening their ecological impact. Acknowledging this medium impact size highlights how businesses may be key players in promoting social and environmental change, which can have far-reaching and long-lasting consequences for sustainable business practices and the general well-being of society.

Given the increased importance placed on “Green Logistics” in the IPMA, businesses should consider taking actions and employing tactics to better capitalize on this factor. The strategic relevance of “Green Logistics” is highlighted in this setting, where it appears as a latent variable with high absolute value. To benefit from its importance, businesses can focus on the following.

- Businesses may concentrate on enhancing and streamlining their supply chain and distribution procedures so that they know how crucial green logistics are. This might be due to the development of energy-efficient warehousing operations, reducing packaging waste, and choosing environmentally friendly transportation solutions.
- Using cutting-edge technological solutions, such as real-time tracking systems and software for route optimization, may significantly enhance the environmental performance of logistics operations. These solutions improve the operating efficiency while simultaneously lowering carbon emissions.
- Green supply chain integration may be facilitated for businesses by forming alliances with suppliers that share their commitment to sustainable operations. Cooperation in environmentally friendly sourcing and materials can decrease the environmental effects of logistics procedures.
- Cultivating a sustainable culture within a company is essential. Green practices can be incorporated into daily operations by offering training and awareness programs to logistics workers.
- Monitoring and evaluating the environmental performance of logistics operations is critical. Companies can monitor progress, define sustainability indicators, and identify development opportunities.
- Committing to green logistics and securing industry certifications while complying with environmental regulations can foster partnerships and agreements.
- Informing consumers about environment-friendly logistical techniques may improve brand loyalty and reputation. Businesses may draw attention to their initiatives to enhance sustainable delivery and reduce their carbon footprints.

Businesses may successfully take advantage of the growing significance of “Green Logistics” by concentrating on these tactics, which will help them achieve sustainability objectives, obtain a competitive edge, and improve their overall environmental responsibility.

## Data Availability

The datasets generated or analyzed during the current study are available from the corresponding author upon reasonable request.

## Declarations

### Guidelines and regulations

All methods were carried out in accordance with relevant guidelines and regulations, ensuring adherence to the highest standards of research ethics.
